# Editorial: Mitochondria in the regulation of anti-tumor immunity

**DOI:** 10.3389/fonc.2025.1596146

**Published:** 2025-04-07

**Authors:** Aqu Alu, Yang Wang, Yuan Cheng

**Affiliations:** ^1^ Department of Biotherapy, Cancer Center, West China Hospital, Sichuan University, Chengdu, China; ^2^ Department of Medical Oncology, National Cancer Center/National Clinical Research Center for Cancer/Cancer Hospital, Chinese Academy of Medical Sciences and Peking Union Medical College, Beijing, China

**Keywords:** tumor, immunity, mitochondria, metabolism, DAMPs

Immunotherapy is one of the most groundbreaking advances in cancer treatment in recent years, offering new hope for many patients. Our Research Topic, *Mitochondria in the Regulation of Anti-Tumor Immunity*, briefly outlines the impact of mitochondrial metabolism and functional abnormalities on tumor immunity. As the powerhouse of the cell, mitochondria serve as a critical bridge connecting tumor metabolism and immune responses by regulating metabolic pathways, releasing damage-associated molecular patterns (DAMPs), and influencing the survival and function of immune cells. In tumor immunotherapy, the function of immune cells such as T cells and natural killer (NK) cells is highly dependent on the metabolic state of mitochondria. For example, the balance between mitochondrial oxidative phosphorylation (OXPHOS) and glycolysis directly affects the activation, differentiation, and persistence of T cells. This topic reviews several studies exploring the role of mitochondria in predicting tumor prognosis, as well as its impact on immunotherapy treatment, aiming to investigate the potential of targeting mitochondria in cancer therapy.

Li et al. explored the mitochondrial metabolism in human papillomavirus (HPV)-associated head and neck squamous cell carcinoma (HNSCC). They detected the OXPHOS levels and glycolysis in four HPV-positive or negative intact cell lines with Seahorse XF Analyzer. High-resolution respirometry in an Oroboros O2K was further used to assess OXPHOS in permeabilized cells. Finally, they performed metabolomic analysis with mass spectroscopy. The results indicated that HPV-positive HNSCC cells exhibit a metabolic shift towards glycolysis rather than OXPHOS while HPV-negative HNSCC showed a more diverse metabolism. This metabolic reprogramming supports cancer cell survival and progression, suggesting that targeting mitochondrial metabolism could be a potential therapeutic strategy for HPV-associated HNSCC in the future.

Xu et al. explored the role of mitochondrial calcium uniporter (MCU) in regulating mitochondrial function and metabolism in gastric cancer (GC). They used the immunohistochemical assay to determine the expression of MCU in the tumor and adjacent tissues of 205 GC patients and applied bioinformatics analysis to analyze its correlation with clinical pathological features and prognosis. Next, they carried out an in-depth study concerning the role of MCU on mitochondrial function, metabolism, biosynthesis, and immune cells. They found that a high expression of MCU was related to poor clinical outcomes of GC patients, characterized by poorer TNM staging and worse survival. Higher MCU expression is associated with altered function of mitochondrial oxidative respiratory chain and metabolism, including nucleotide, amino acid, and fatty acid synthesis.

Similarly, Zhao et al. find that overexpression of DNM1L (a key regulator of mitochondrial fission) is closely related to poor overall survival in GC patients. COX regression analysis indicated that DNM1L is an independent prognosis factor, making it a potential biomarker for GC patients. Bioinformatics and CIBERSORT analysis suggested that DNM1L is related to various signaling pathways and immune responses, altering immune cell infiltration in tumor tissues. Thus, targeting mitochondrial proteins could be a novel and promising treatment strategy. Wang et al. pointed out that mitochondrial dysfunction contributes to the failure of multiple immune cells, including T cells, NK cells, dendritic cells, etc., leading to immune evasion by inhibiting immune activation and creating immunosuppressive tumor microenvironment. This makes restoring mitochondrial health essential for the stimulation of effective antitumor immune responses.

In summary, this Research Topic promoted the research progress in the field of mitochondrial and anti-tumor immunity regulation, highlighting the indispensable role of dysfunctional mitochondria in cancer progression and immune evasion, leading to poor clinical outcomes. Therefore, targeting mitochondria has become a promising and novel strategy to fight tumors. Future researches need to focus on the in-depth study of the mechanism of mitochondria affecting anti-tumor immunity, as well as the development of drugs or therapeutic measures targeting mitochondria, to promote the translation of research into clinical practice, and finally benefit cancer patients.

